# Interacting Microbe and Litter Quality Controls on Litter Decomposition: A Modeling Analysis

**DOI:** 10.1371/journal.pone.0108769

**Published:** 2014-09-29

**Authors:** Daryl Moorhead, Gwenaëlle Lashermes, Sylvie Recous, Isabelle Bertrand

**Affiliations:** 1 Department of Environmental Sciences, University of Toledo, Toledo, Ohio, United States of America; 2 INRA, UMR614 Fractionnement des AgroRessources et Environnement, Reims, France; 3 Université Reims-Champagne Ardenne, UMR614 Fractionnement des AgroRessources et Environnement, Reims, France; NERC Centre for Ecology & Hydrology, United Kingdom

## Abstract

The decomposition of plant litter in soil is a dynamic process during which substrate chemistry and microbial controls interact. We more clearly quantify these controls with a revised version of the Guild-based Decomposition Model (GDM) in which we used a reverse Michaelis-Menten approach to simulate short-term (112 days) decomposition of roots from four genotypes of *Zea mays* that differed primarily in lignin chemistry. A co-metabolic relationship between the degradation of lignin and holocellulose (cellulose+hemicellulose) fractions of litter showed that the reduction in decay rate with increasing lignin concentration (LCI) was related to the level of arabinan substitutions in arabinoxylan chains (i.e., arabinan to xylan or A∶X ratio) and the extent to which hemicellulose chains are cross-linked with lignin in plant cell walls. This pattern was consistent between genotypes and during progressive decomposition within each genotype. Moreover, decay rates were controlled by these cross-linkages from the start of decomposition. We also discovered it necessary to divide the Van Soest soluble (labile) fraction of litter C into two pools: one that rapidly decomposed and a second that was more persistent. Simulated microbial production was consistent with recent studies suggesting that more rapidly decomposing materials can generate greater amounts of potentially recalcitrant microbial products despite the rapid loss of litter mass. Sensitivity analyses failed to identify any model parameter that consistently explained a large proportion of model variation, suggesting that feedback controls between litter quality and microbial activity in the reverse Michaelis-Menten approach resulted in stable model behavior. Model extrapolations to an independent set of data, derived from the decomposition of 12 different genotypes of maize roots, averaged within <3% of observed respiration rates and total CO_2_ efflux over 112 days.

## Introduction

Recent studies are challenging the ways that we have traditionally perceived and modeled decomposition. Decomposers were once thought to rapidly degrade labile fractions of plant litter, such as carbohydrates and proteins, leaving more recalcitrant compounds, like lignin, to become the foundation of stabilized soil organic matter [1], [2], [3]. However, microbial products rather than plant lignin actually may represent the larger fraction of SOM [4], [5], and lignin *per se* may not persist throughout decay [6]. Unfortunately, many mathematical models are based on the traditional view of decomposition. In part this is because the chemical composition of litter has often been evaluated by proximate carbon analysis, typically yielding three, qualitatively different pools of compounds: polar and nonpolar extractives, acid hydrolysable materials, and acid non-hydrolysable materials. These pools provide a convenient structural framework for modeling, but lack the resolution to address finer scale biochemical transformations revealed by more contemporary studies [4], [5], [6]. Mathematical models must change to reflect these observations.

One of the changes needed in traditional decomposition models is to explicitly simulate the activities of microorganisms. If microbial contributions to SOM are more substantial than lignin, then the litter compounds fueling microbial activity may be more important to C stabilization than the plant lignin pool. This point was suggested by Smith et al. [7] who noted that the proportion of initial litter C remaining over time (125 days) was directly related to the initial rate of decomposition prior to lignin decay. In other words, the incorporation of C into microbial biomass increased its overall persistence despite an initially higher fractional loss through respiration [2], [8]. A simple explanation is that the higher carbon use efficiency (CUE) of readily decayed materials, like sugars and proteins, transfers a larger fraction of substrate C to microbial biomass than lignin, and that these microbial products are more persistent [4], [5]. These results suggest that the early stage of litter decay when microbial activities are greatest should be an integral component of mathematical models.

A second change needed in decomposition models is how they respond to differences and interactions between the chemical constituents of decaying litter. Despite the questionable role of plant lignin in decomposition, both the initial lignin content and the lignocellulose index (LCI) of litter are often the best predictors of decay rate [1], [3]. The lignocellulose index is normally calculated as the ratio of the acid non-hydrolysable/(non-hydrolysable+hydrolysable) products of proximate C analysis (see above), and assumed to represent the lignin (non-hydrolysable) and holocellulose (hydrolysable) fractions of litter. Although this assumption may be nearly true for fresh litter, both microbial and lignin degradation products increasingly contribute to proximate C fractions during decay [5], [6]. Thus LCI and proximate C fractions are ambiguous metrics of litter chemistry and models using them to regulate decomposition conflate litter quality controls with the decomposition process. In reality, plant cell walls are usually the largest component of plant litter and have a specific biophysical structure of interconnected saccharide and phenolic polymers [9]. This arrangement partly explains the relatively consistent relationship between LCI and decay, but raises questions about the precise relationships between lignin and other litter constituents [10], [11], [12] needed to more accurately model substrate dynamics during decomposition.

Few empirical studies have examined litter chemistry during decay with sufficient resolution to improve models beyond the lignin or LCI controls normally calculated. However, Machinet et al. [11], [13], [14] examined the detailed litter chemistry of maize (*Zea mays* L.) roots for naturally occurring genotypes decomposing in laboratory incubations. These genotypes varied primarily in their lignin content. Long-term C losses (>200 days) were most often correlated with compounds associated with lignin and cross-linked between lignin and polysaccharides, whereas short-term (<200 days) losses were more closely related to soluble compounds and cell wall sugars [11]. Different loss rates of different sugars (e.g., glucans, xylans and arabinans) suggested that the relationship between lignin and decomposition was partly mediated by the specific composition of the polysaccharide fraction of the litter, probably because arabinan substitution in xylan chains interferes with the degradation of hemicellulose [9]. In other words, the sugar composition of cell walls appears to define the transition from short- to long-term patterns of decay, providing a mechanistic explanations for patterns in microbial activity, as well as the empirical relationships between LCI and decay most often used in decomposition models [15].

The overall goal of this study was to simulate the relationships between litter decay, microbial production and litter chemistry at the early stage of decomposition, using data collected by Machinet et al. [11], [13] to test and refine the Guild Decomposition Model (GDM) developed by Moorhead and Sinsabaugh [16]. Our two specific objectives were to simulate patterns of microbial productivity during decomposition as litter chemistry changed, and to simulate the relationship between measured changes in cell wall chemistry (sugars and lignin) and decay rate. We chose GDM because it calculates the decay of specific substrate pools as a combined function of both microbial activity and substrate characteristics, using the Michaelis-Menten equation of substrate saturation. The maximum velocity of decomposition (V_max_) is thus a function of microbial biomass [16], so that estimated decay rates reflect both the changing chemical composition of the decomposing litter and microbial productivity.

## Methods

Our general approach was to revise GDM to use the empirical data collected by Machinet et al. [11], [13], [14] to derive parameters needed to simulate decomposition. Then, we analyzed the data from a 112-day laboratory study of four maize genotypes that differed from one another in initial litter chemistry [13], [14], to evaluate interactions between litter qualities likely controlling the decomposition process, and to obtain estimates for parameter values used to describe these processes. Next, we selected two key parameters for GDM, which preliminary analyses indicated varied with initial litter chemistry and had large effects on model behavior (below). We then optimized these parameters to produce the best possible fit between simulated and observed patterns of decomposition, with respect to CO_2_ efflux and chemical transformations in decaying litter. Best-fit parameter values were in turn compared to initial litter chemical characteristics to determine possible relationships. These relationships were then used to derive parameter values needed to simulate CO_2_ efflux during decomposition of maize roots for 12 additional genotypes [11].

### Experimental data

The data used to drive the revised GDM model were obtained from Machinet et al. [13]. Briefly fine roots (diameter 2–3 mm) of four natural genotypes of maize (F2, F2bm1, F292 and F292bm3), which differed in their chemical composition (Table 1), were cut into 5 mm lengths, added to soil in laboratory microcosms and incubated at 15°C. Potassium-nitrate fertilizer was added to microcosms to insure no nitrogen limitation [17]. They monitored CO_2_-C efflux on days 3, 7, 10, 14, 21, 29, 36, 42, 51, 57, 70, 80, 87, 95 and 112. Chemical characteristics of litter were determined on days 0 (initial), 14, 36, 57 and 112, on roots manually removed from soils. Machinet et al. [11] used the same experimental methods for 12 additional maize genotypes, but examined only the initial litter chemistry and measured CO_2_ efflux during decomposition.

**Table 1:** Model parameters and state variables.

Factor	Description	Values^a^ (genotype)	Unit
k_1max_	Decay rate coefficient of substrate C_1_	0.1	day^−1^
k_2max_	Maximum decay rate coefficient of substrate C_2_	0.047^b^	day^−1^
k_3max_	Maximum decay rate coefficient of substrate C_3_	0.001	day^−1^
e_1_	Substrate C_1_ use efficiency	0.4	unitless
e_2_	Substrate C_2_ use efficiency	0.3	unitless
g	Coefficient for basal respiration	0.001	day^−1^
BC_max_	Maximum Biomass: total C ratio	0.05	unitless
LCI_T_	LCI value at which C_3_ decay starts	0.42 (F2)	unitless
		0.36 (F2bm1)	
		0.46 (F292)	
		0.45 (F292bm3)	
LCI_max_	LCI value at which decay stops	0.7	unitless
K_Bj1_	Half-saturation coefficient of all Guilds for substrate C_1_	10	mg C·kg^−1^ soil
K_B22_	Half-saturation coefficient of Guild 2 for	29 (F2)	mg C·kg^−1^
	substrate C_2_	33 (F2bm1)	soil
		23 (F292)	
		19 (F292bm3)	
K_B32_	Half-saturation coefficient of Guild 3 for	300	mg C·kg^−1^
	substrate C_2_		soil
K_B33_	Half-saturation coefficient of Guild 3 for substrate C_3_	500	mg C·kg^−1^ soil
C_1T_	Non-decomposable fraction of C_1_	0.61^b^ (F2)	unitless
		0.60 (F2bm1)	
		0.71 (F292)	
		0.90 (F292bm3)	
C_1_	Litter C in C_1_ pool (Van Soest soluble)	364 (F2)	mg C·kg^−1^
		419 (F2bm1)	soil
		361 (F292)	
		288 (F292bm3)	
C_2_	Litter C in C_2_ pool (acid hydrolysable fraction)	1238 (F2)	mg C kg^−1^
		1166 (F2bm1)	soil
		1295 (F292)	
		1387 (F292bm3)	
C_3_	Litter C in C_3_ pool (acid non-hydrolysable	397 (F2)	mg C·kg^−1^
	fraction)	415 (F2bm1)	soil
		344 (F292)	
		325 (F292bm3)	
B_1_	C in Guild 1	10	mg C·kg^−1^
			soil
B_2_	C in Guild 2	10	mg C·kg^−1^
			soil
B_3_	C in Guild 3	5	mg C·kg^−1^
			soil

a Initial values for variables.

b Empirically determined from experimental data.

The suite of litter chemical characteristics reported by Machinet et al. [11], [13] in root residues included C and N content, Van Soest soluble C (C-SOL) and N (N-SOL) contents, and cell wall polysaccharides: glucan (Glu), arabinan (Ara) and xylan (Xyl), which are the major polymer carbohydrates in graminaea, as well as galactan (Gal) and uronic acids contents (galacturonic (Ac Gal) and glucuronic (Ac Glu)). They also determined Klason lignin (KL) and the lignin monomers, guaiacyl (G) and syringyl (S), as well as ester-linked *p*-coumaric (pCA) and ferulic acids (FA_ester_), and ether-linked ferulic acids (FA_ether_). From these data we calculated the sum of cell wall sugars (∑Sug), arabinan∶xylan ratio (A∶X), carbon∶nitrogen ratio (C∶N), lignocellulose index (LCI = C_3_/[C_2_+C_3_])) with C_1_ ( = C-SOL), C_2_ ( = ∑Sug) and C_3_ ( = KL), and non-detergent fiber (NDF) content.

Machinet et al. [13] estimated mass loss of litter based on cumulative CO_2_ carbon efflux. Concentrations of litter chemical fractions at each date were multiplied by the estimated mass of remaining litter to estimate pool sizes during decomposition. Decay rate coefficients (k_i_) were calculated for chemical constituents C_1_, C_2_ and C_3_ for all 4 litter types over all 4 periods of observation (days 0–14, 14–36, 36–57 and 57–112), as the difference in the natural log of pool size between observation dates, divided by the time period.

### Model revisions

**Figure 1:**
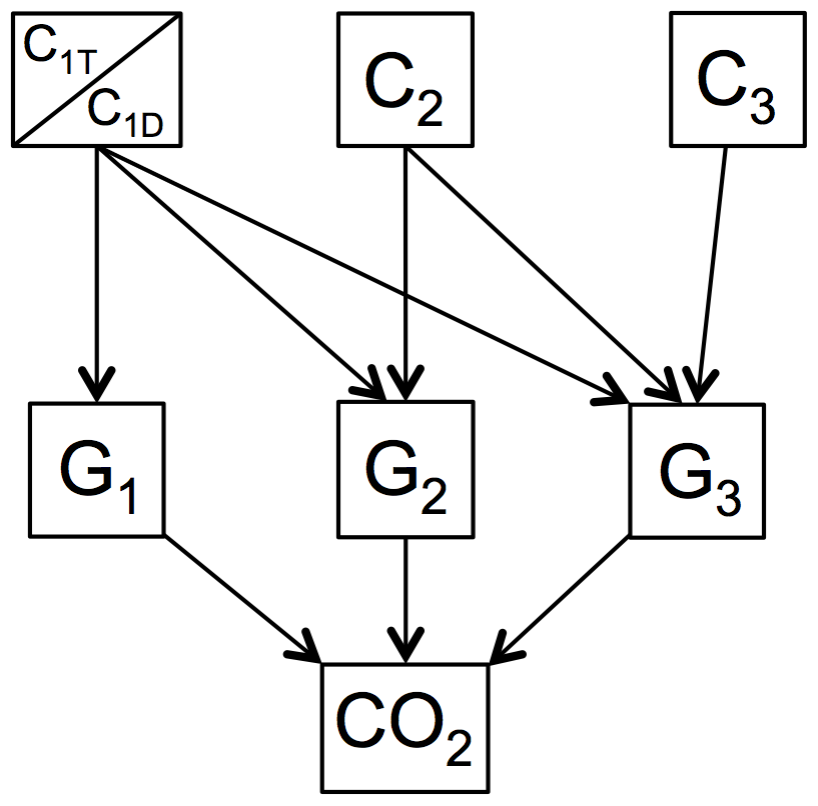
Carbon flow diagram for revised Guild Decomposition Model (GDM) simulating *Zea mays* root decomposition. Litter pools are two Van Soest soluble fractions, a decomposable fraction (C_1D_) and a resistant fraction (C_1T_), acid hydrolysable (C_2_) and acid non-hydrolysable (C_3_) fractions, and microbial guilds of opportunists (G_1_), cellulolytic decomposers (G_2_) and lignolytic decomposers (G_3_).

This model was programmed in MATLAB (The MathWorks Inc., Natick, USA). We revised GDM to use Reverse Michaelis-Menten (RMM) functions to calculate decay rates as functions of microbial activity [18]: dC_i_/dt = k_i_·C_i_·B_j_/(K_Bji_+B_j_), where C_i_ is the amount of substrate *i*, B_j_ is the amount of microbial biomass in guild *j*, and K_Bji_ is the half-saturation coefficient of guild *j* for substrate *i*. Note that we altered the more common RMM approach by using biomass (B) in place of an enzyme (E) pool, which assumes a constant ratio of E∶B resulting from constitutive enzyme production [16], [18]. We limited access to substrate pools by guilds: guild 1 was assumed to access only soluble resources (C_1_); guild 2 was assumed to access both C_1_ and C_2_; guild 3 could access C_1_, C_2_ and C_3_ (Figure 1).

A final revision to GDM was necessary to capture the dynamics of the soluble pool (C_1_), a fraction of which persisted throughout the study by Machinet et al. [13]. This persistent fraction (C_1T_) varied between genotypes and was considered to be non-decomposable during the time frame of the study (112 days).

### Parameters and state variables

All parameters and state variables are reported in Table 1. Values of C_1T_, the persistent fraction of the initial C_1_ pool, were estimated from the average values of C_1_ over time for each genotype (Table 1). We selected a decay rate coefficient (k_1_) of 0.1 for the decomposable fraction of the C_1_ pool (C_1D_), due to its rapid loss.

**Figure 2:**
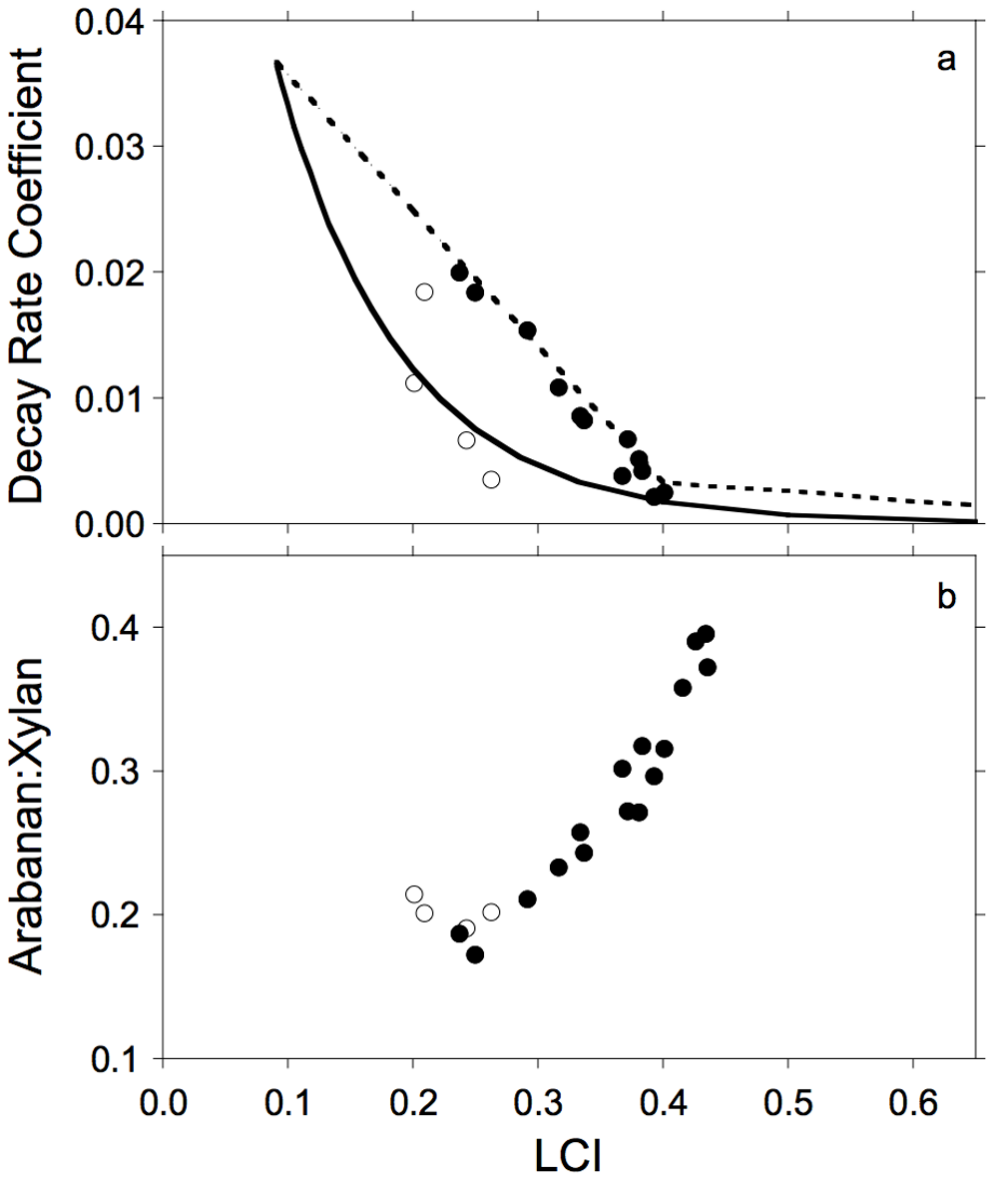
Observed and simulated decay rate coefficients for holocellulose and araban∶xylan composition of remaining litter at residual values of lignocellulose index (LCI). Relationships between observed and simulated: a. decay rate coefficients (k_2_) for litter pool C_2_ and lignocellulose index (LCI) of litter (solid line is simulated according to Moorhead and Sinsabaugh [16], dashed line is simulated according to Moorhead et al. [19]), b. relationship between arabinan∶xylan (A∶X) and LCI contents of decaying litter. Open circles are observations based on initial litter chemistry (day 0) and solid circles are from observations over time.

The RMM approach made it possible to simplify the balance of C_2_ and C_3_ decay as functions of lignocellulose index (LCI) according to Moorhead et al. [19] (Text S1). In brief, the decay rate coefficients (k_2_ and k_3_) were described as linear functions of LCI: k_i_ = m_i_·LCI+k_imax_, given empirically observed maximum values (k_imax_) and slopes (m_i_). This approach assumes that k_3_ = 0 at a threshold level of LCI = LCI_T_, wherein LCI_T_ = 0.4 [20], which defines the point at which LCI changes from being solely determined by C_2_ decay (LCI≤LCI_T_) to also being determined by C_3_ decay (LCI>LCI_T_). The value of k_2max_ (maximum coefficient of C_2_ decay) was estimated as the intercept of the linear regression of the observed decay rate coefficients for C_2_ (k_2_) against litter LCI for the four maize genotypes (Figure 2a; Table 1) over days 14–112, excluding values estimated over days 0–14 when we assumed that microorganisms had not begun to fully utilize pool C_2_. Values of k_2_ used during simulations were then estimated according to LCI of remaining litter. This revision to GDM provided a closer fit to observed patterns of C_2_ decay (Figure 2a; N = 12, R^2^ = 0.97, P<0.01) than Moorhead and Sinsabaugh [16]. The value of k_3max_ was set to 0.001 because there was little evidence of C_3_ decomposition for any litter type during incubations [13].

We selected identical values of K_B11_, K_B12_ and K_B13_, the half-saturation coefficients for the utilization of pool C_1_ by all three guilds, assuming all microorganisms have similar affinities for soluble substrates (Table 1). We initially set K_B22_ and K_B32_ at the same values, again assuming that organisms capable of using the resource would have similar half-saturation coefficients, but higher than those for C_1_, assuming that C_2_ was generally less decomposable than C_1_. We then selected an even higher value for K_B33_ assuming that C_3_ was even less decomposable than C_2_. We are unaware of any published values for these parameters, and so followed the rationale of Moorhead and Sinsabaugh [16] in choosing values reflecting relative access to substrates of different qualities.

GDM requires an estimate of initial decomposer biomass, which it divides among three distinct microbial pools: early opportunists (guild 1), subsequent decomposition specialists (guild 2) and a final group of lignin degraders (guild 3) (Figure 1). Machinet et al. [13] estimated that microorganisms colonizing maize roots contributed 0–11% of the initial litter carbon content, and other studies suggest that microbial biomass rarely exceeds 2–3% of soil organic matter [21], [22]. We set the initial microbial biomass pool at 1.25% (25 mgC·kg^−1^ soil) of total litter mass, assuming 10 mgC·kg^−1^ soil for guilds 1 and 2, and 5 mgC kg^−1^ soil for guild 3 [16].

We estimated microbial production as the difference between the quantity of carbon released from decaying substrates and the amount mineralized through both growth- and maintenance-associated respiration. We assumed that this difference was immobilized in microbial biomass. GDM also calculates microbial turnover necessary to keep total microbial C less than 5% of the total system's organic C (microorganisms+substrates; Table 1). We estimated carbon use efficiency (CUE) as the difference between the amounts of C released from decaying substrates and mineralized through respiration, divided by the amount of C released by decomposition [23].

### Optimizing parameter values

The values of two key model parameters that showed correlations with initial chemistry, LCI_T_, and K_B22_, were optimized for the four maize genotypes [13] using the *fmincon* function of the MATLAB Optimization Toolbox (The MathWorks, Inc., Natick, USA), which applies a sequential quadratic programming algorithm. The objective function was the sum of the root mean square errors calculated between the experimental and simulated CO_2_ efflux rates, cumulative amount of carbon mineralized over 112-days and chemical evolution in the C_1_ and C_2_ pools during decomposition, normalized by the means of the observations.

We determined the best-fit estimates of parameters K_B22_ and LCI_T_ that most closely matched simulations to observed patterns of CO_2_ efflux and both C_1_ and C_2_ pools over time (Table 1). We selected these parameters because the dynamics of pool C_2_ were most closely related to cumulative CO_2_ efflux and thus, estimated mass loss.

### Sensitivity analysis

To evaluate the sensitivity of the model to parameter estimates (Table 1), we randomly varied model parameters e_1_, e_2_, k_1max_, BC_max_ and K_B11_ within ±10% of their initial values (Table 1), 100 times for each litter type. We then calculated the differences between simulated and observed values of C_1_, C_2_, cumulative CO_2_, and respiration rates on each date of observation [13] as a relative value = (observation-simulation)/observation. We summed these relative differences for each type of observation over all days of observation (i.e., through day 112), which produced a composite measure of the relative differences between observations and model output for each maize genotype. ANCOVA evaluated the contributions of variations in parameter values to variations in the relative differences between model output and observations by litter type. The type II sums of squares from ANCOVA were interpreted to represent the relative contribution of each parameter to model behavior.

### Model extrapolation

The last set of simulations estimated decomposition for the 12 additional maize genotypes examined by Machinet et al. [13]. Relationships between values of C_1T_ and best-fit values of LCI_T_ and K_B22_ (Table 1) and the initial characteristics of litter chemistry were estimated for the four litter types described by Machinet et al. [13]: LCI_T_ = −0.36·KL/AX+0.70 (N = 4; R^2^ = 0.923; P≤0.05); C_1T_ = 0.02 · ∑Sug – 0.64 (N = 4; R^2^ = 0.998; P≤0.05); and K_B22_ = 53.22 · KL/AX −13.65 (N = 4; R^2^ = 0.975; P≤0.05). These relationships were then used to estimate parameter values of C_1T_, LCI_T_ and K_B22_ for simulations with each of the 12 additional genotypes reported by Machinet et al. [11], based on their initial chemistry. Principal components analyses evaluated relationships between the relative differences in observed and simulated values of respiration rates and cumulative CO_2_ efflux, and initial litter chemistry characteristics for all 12 litters to determine if more detailed litter chemical characteristics than currently used in the model could provide additional insights to litter quality controls on decomposition.

## Results

### Decomposition dynamics

We discovered a close correspondence between the arabinan∶xylan ratio (A∶X) and lignocellulose index (LCI) of the residue (Figure 2b) in the empirical data [11]. Between days 14 and 112, a linear regression of AX over LCI yielded an R^2^ = 0.91 (N = 12, P≤0.01); we omitted days 0–14 because we expected decomposition to be limited by microbial activity rather than substrate [16], [18]. Perhaps this relationship also explains why both AX and KL were related to best-fit model parameters LCI_T_ and K_B22_.

**Figure 3:**
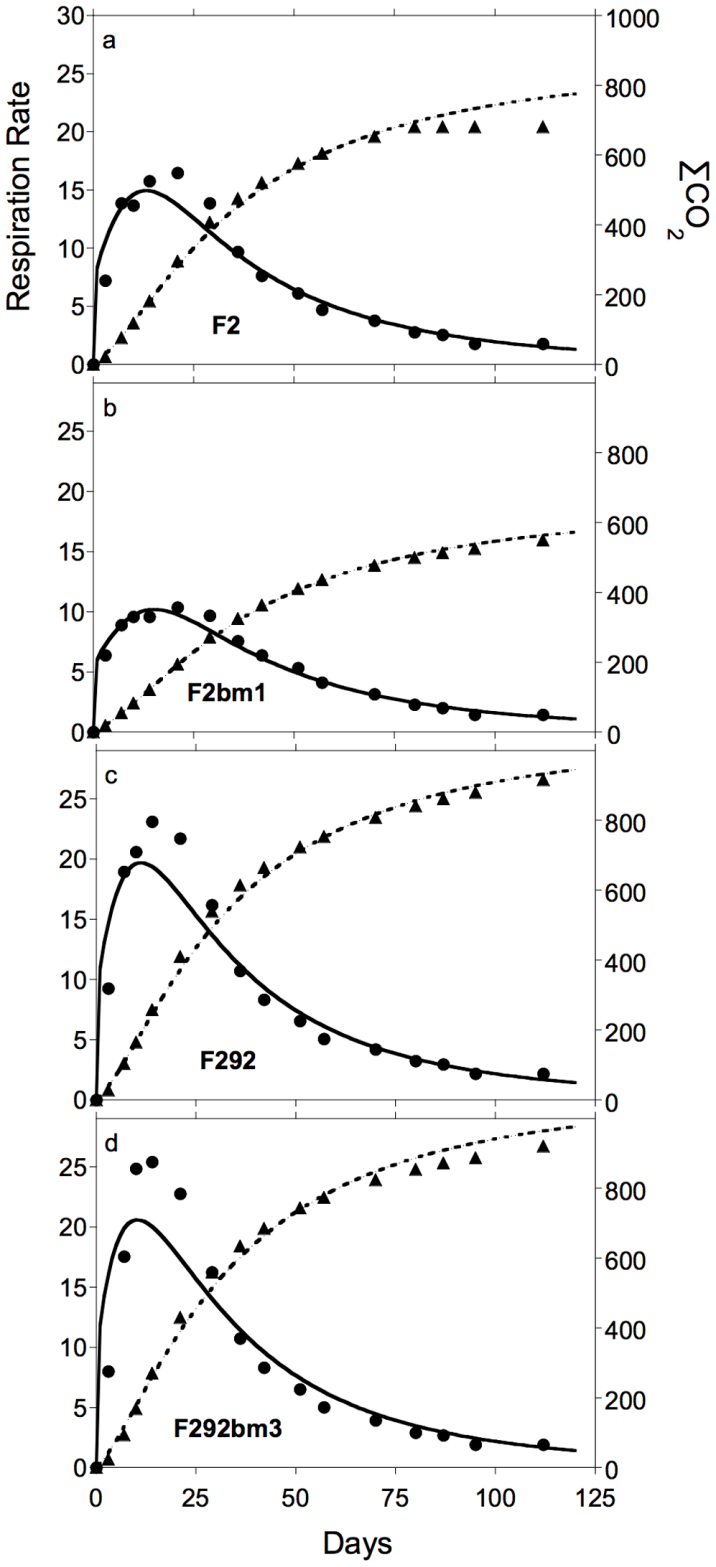
Observed and simulated respiration rates and cumulative CO_2_ efflux during decomposition of *Zea mays* root litter. Observed (symbols) and simulated (lines) patterns of respiration rates (solid lines and circles, mgC·kg soil^−1^·d^−1^) and cumulative CO_2_ efflux (dashed lines and triangles, mgC·kg soil^−1^) for maize mutants, a. F2, b. F2bm1, c. F292 and d. F292bm3.

**Figure 4:**
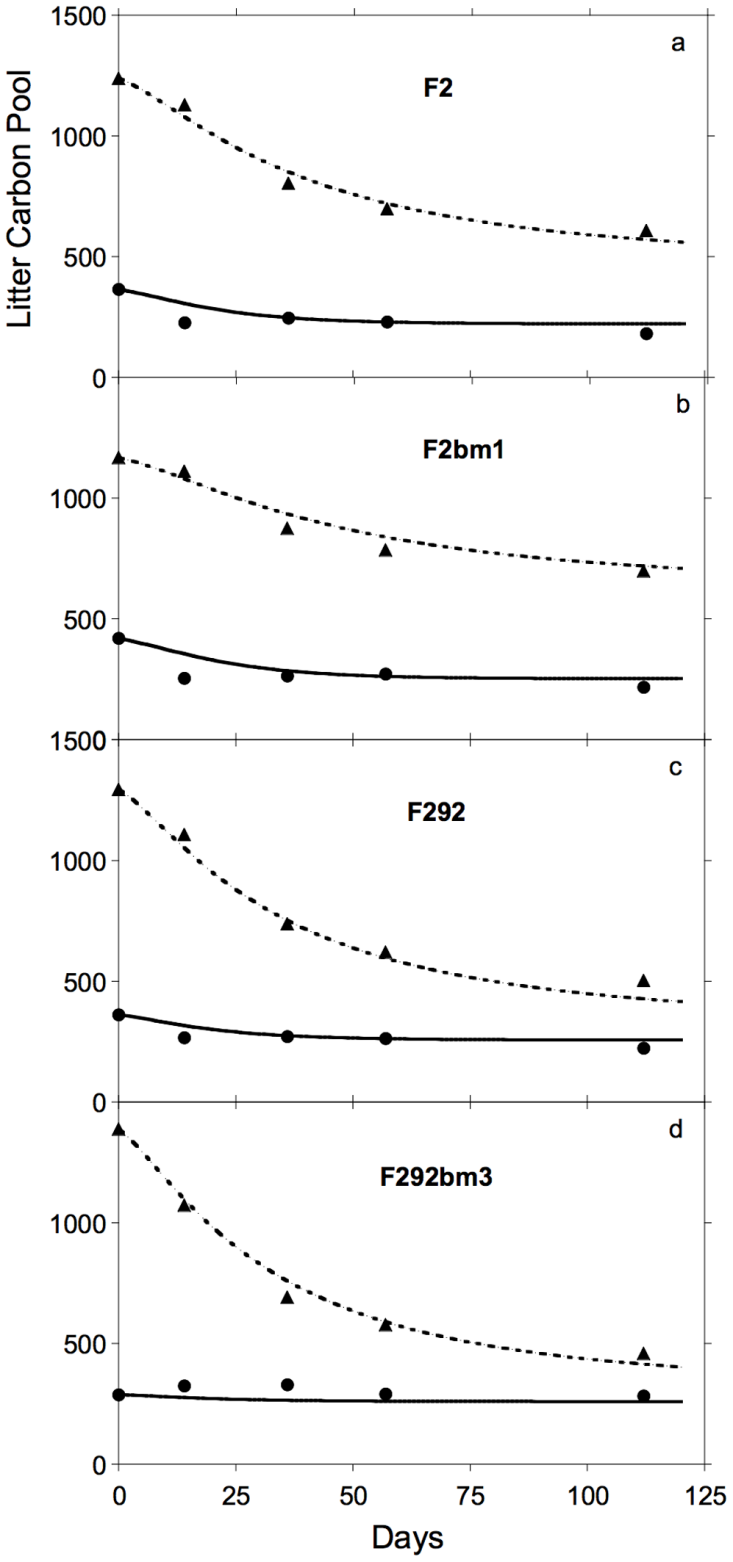
Observed and simulated mass remaining for soluble (C_1_) and acid hydrolysable (C_2_) chemical fractions of litter during decomposition of *Zea mays* roots. Observed (symbols) and simulated (lines) patterns of mass remaining for soluble C_1_ pool (solid lines and circles, mgC·kg soil^−1^) and acid hydrolysable C_2_ pool (dashed lines and triangles, mgC·kg soil^−1^) during decomposition of litter from maize mutants, a. F2, b. F2bm1, c. F292 and d. F292bm3.

Rates of CO_2_ efflux for all four litter types rapidly increased to peak values within 14–21 days followed by gradual declines (Figure 3); cumulative CO_2_ efflux rose rapidly during the first 36 days and then more slowly until day 112. For 3 of the 4 genotypes, the C_1_ pool declined rapidly from day 1 to day 14, and then remained relatively constant during the rest of the incubation (Figure 4). For genotype F292bm3, the pool of C_1_ remained essentially unchanged throughout the study. The C_2_ pools of all four litters declined throughout the incubations, accounting for most of the litter mass loss over time (Figure 4). The C_3_ fraction of the remaining litter showed a slight increase over the first 14 days of incubation (ca. 5–10%) for both F2 genotypes (not shown), but remained roughly constant for the two F292 genotypes [13].

### Microbial production

**Figure 5:**
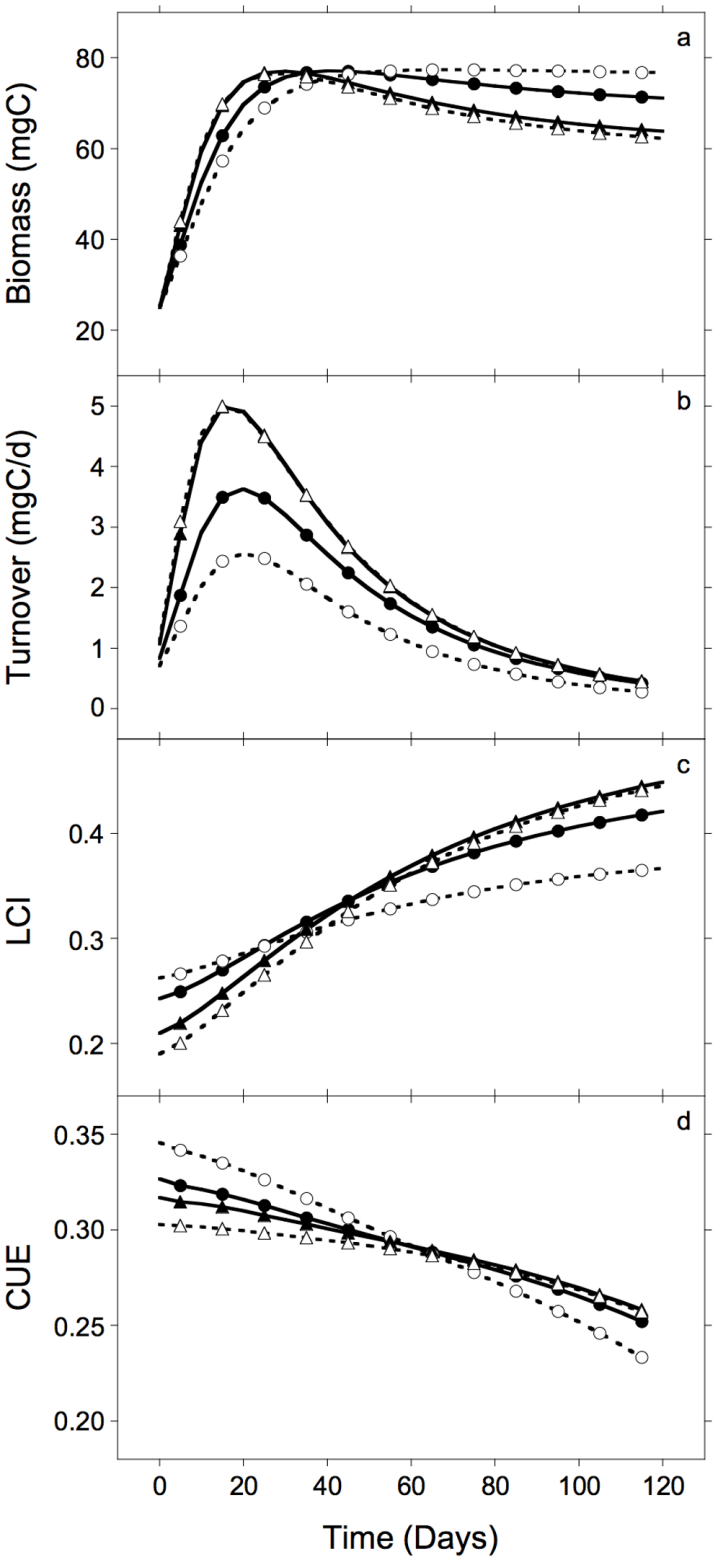
Simulated values of microbial biomass and turnover rate, remaining litter lignocellulose index (LCI), and carbon use efficiency (CUE), during decomposition of *Zea mays* root litter. Simulated values of a. total microbial biomass, b. daily microbial turnover rate, c. remaining litter lignocellulose index (LCI), and d. realized carbon use efficiency (CUE), over time, for litter genotypes F2 (filled circles), F2bm1 (open circles), F292 (filled triangles) and F292bm3 (open triangles).

Microbial biomass rapidly increased to peak values within 20–40 days, varying among genotypes (Figure 5a), declining most rapidly for those genotypes supporting the most rapid initial growth with the highest decay rates (F292 and F292bm3). Microbial turnover rates (Figure 5b), which should correlate with the generation of microbial products such as cell walls, were similar in shape but had lower peak values and lagged behind the patterns of rising and falling respiration rates (Figure 3). Genotypes with higher turnover rates also showed the greatest declines in biomass by day 112. Litter LCI increased over time in all litter types, coincident with declining biomass and turnover rates (Figure 5c), increasing most rapidly for litters decaying most rapidly (Figure 3). CUE declined over time for all litter types (Figure 5d), coincident with increasing LCI (Figure 5c), but started at higher values in litter types with larger pools of labile C_1_ (i.e., C_1D_), which had a higher C-assimilation efficiency (e_i_) than C_2_ (Table 1).

The total microbial production (C-immobilized into biomass including microbial turnover) by day 112 varied between litter types: 248, 195, 295 and 295 mgC for genotypes F2, F2bm1, F292 and F292bm3, respectively. These values were negatively related to initial litter KL and KL/AX contents, as well as final biomass and best-fit values of K_B22_. They were positively related to total cumulative CO_2_ efflux by day 112 and best-fit values of LCI_T_ (all N = 4, P≤0.05).

### Model test

Our initial model parameter set, including best-fit estimates of K_B22_ and LCI_T_, simulated rates of CO_2_ efflux closely matching observations (all N = 15; omitting day 0), with R^2^ values ranging from 0.87 for genotype F2bm3 to 0.98 for genotype F2bm1 (Fig. 3). Simulated rates peaked before observations for all litter types and at slightly lower values. However, simulated peak rates were within 10% of observed peaks for both F2 genotypes and 20% for both F292 genotypes. Simulated values of cumulative CO_2_ efflux (to day 112) were even closer to observations, with R^2^ values in excess of 0.99 for all genotypes (Figure 3). All simulations were within 5% of observed cumulative CO_2_ efflux values. Simulated values of C_2_ seldom differed by more than 5% of the observed values, with all R^2^≥0.99 (Figure 3). Finally, our model did not estimate any loss in pool C_3_ during simulations and has no mechanism to generate an increase in this pool's size (Figure 1).

### Sensitivity analysis

The relative contributions of individual parameters to explaining variations in model behaviors differed by litter type and observation (Table 2). For example, the efficiency of C assimilation from substrate C_1_ (e_1_) rarely explained more than 1% of the variation in model behavior for any litter type. Moreover, no single parameter made a substantial contribution (≥10%) to explaining the variation in any model behavior when all four genotypes were pooled (not shown). When litter types were examined separately, variations in k_1max_ and K_B11_ often made their largest contributions to the same output variables for the same litter types. For example both parameters made substantial contributions (≥10%) to variations in C_1_ for litter types F2, F2bm1 and F292bm3; cumulative CO_2_ efflux for litter types F2, F2bm1 and F292; respiration rates for litter types F2bm1 and F292; and overall model fit for litter type F2bm1. Neither parameter made a substantial contribution to variation in C_2_ for any litter type. In contrast, parameters e_2_ and BC_max_ often made their largest contributions to model behaviors for litter types when k_1max_ and K_B11_ did not. For example, e_2_ and BC_max_ made substantial contributions to variations in C_2_ for litter types F2, F2bm1 and F292bm3; cumulative CO_2_ efflux for litters F292 and F292bm1; respiration rates for F2, F292 and F292bm3; and overall model fit for F2.

**Table 2:** Results of sensitivity analysis quantifying the relative contributions (%) of random variations in model parameters (column headings) to resulting variations in model behaviors (row labels), based on sums of squares from ANOVA relating model output to parameters.

Source	Genotype	e_1_	e_2_	k_1max_	BC_max_	K_B11_
C_1_-Carbon	F2	0.2*	1.3*	47.5**	3.9*	30.6**
C_1_-Carbon	F2bm1	2.0*	0.5*	63.7**	0.7*	38.9**
C_1_-Carbon	F292	0.1*	1.7	3.9*	42.5**	1.2
C_1_-Carbon	F292bm3	0.0	2.3*	65.6**	7.0*	22.1**
C_1_-Carbon	All Litters	0.2	0.0	2.1*	0.1	6.3*
C_2_-Carbon	F2	1.1*	38.4**	0.2*	53.9**	0.1
C_2_-Carbon	F2bm1	1.2	13.2**	0.1*	71.4**	0.0
C_2_-Carbon	F292	0.0	3.5*	0.8*	73.0**	0.1
C_2_-Carbon	F292bm3	0.0	40.8**	0.0	40.9**	0.0
C_2_-Carbon	All Litters	1.0*	1.6*	0.2	3.3*	0.4
Cumulative CO_2_	F2	0.2*	1.2*	48.1**	3.8*	30.1**
Cumulative CO_2_	F2bm1	0.4*	0.1	64.4**	0.2*	40.3**
Cumulative CO_2_	F292	0.7*	11.7**	35.7**	12.3**	38.1**
Cumulative CO_2_	F292bm3	0.5*	48.8**	6.6*	21.2**	4.5*
Cumulative CO_2_	All Litters	0.2	0.0	0.6	0.3	1.6*
Respiration Rate	F2	1.0	28.7**	9.6*	17.8**	7.6*
Respiration Rate	F2bm1	0.3	0.1	48.5**	0.3	30.2**
Respiration Rate	F292	2.1*	40.8**	20.6**	11.4**	14.0**
Respiration Rate	F292bm3	0.2*	45.3**	1.8*	34.7**	0.5*
Respiration Rate	All Litters	0.0	0.2	0.0	0.4	0.1
Overall Fit	F2	3.0*	34.8**	4.0*	31.9**	2.1*
Overall Fit	F2bm1	0.6*	0.2	57.9**	0.6*	36.3**
Overall Fit	F292	2.6*	46.5**	4.6*	16.8*	7.9*
Overall Fit	F292bm3	0.3*	49.2**	0.9*	31.3*	0.7*
Overall Fit	All Litters	0.0	0.2	0.6	0.4	0.6
Maximum		30.4	49.2	65.6	73.0	40.3
Count>10%		0	11	9	12	9

*significant parameter contributions (P≤0.05) to variation in model behavior.

**contributions of parameters representing at least 10% of the explained variation in model behavior (P≥0.05).

### Model extrapolation

**Figure 6:**
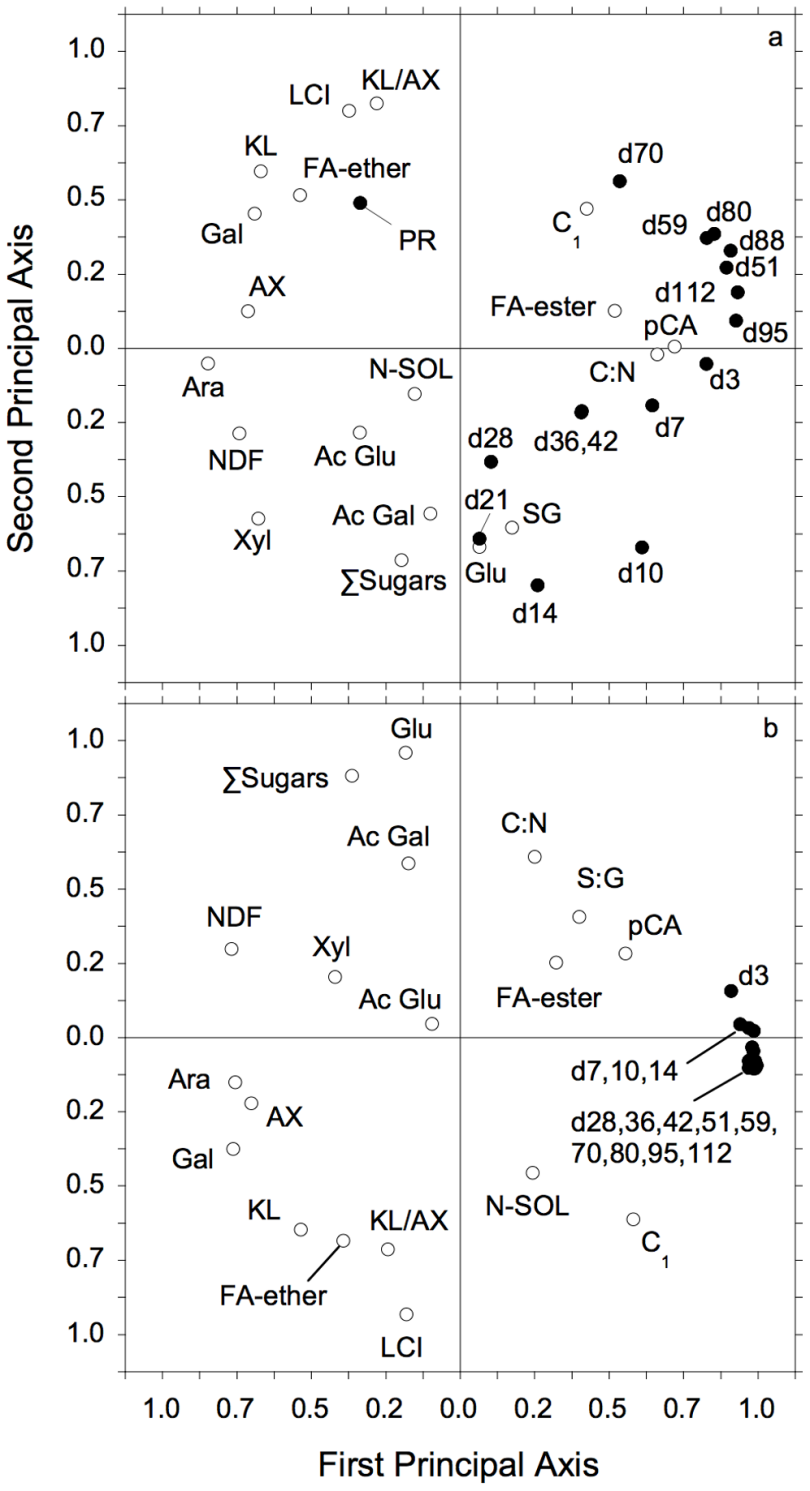
Principal components analysis of variations in initial litter chemistry characteristics and relative differences between observed and simulated rates of microbial respiration and cumulative CO_2_ efflux during decomposition of *Zea mays* roots. Results of principal components analysis of variations in initial litter chemistry characteristics and relative differences = (observations – simulations)/observations from simulated decomposition of 12 novel maize mutants [11] for: a. rates of respiration on days 3–112 (e.g., d3 = rate on day 3) and peak respiration (PR), and b. cumulative CO_2_ efflux by days 3–112 (parameter definitions given in text).

When we simulated decomposition of the 12 different maize genotypes [11], we found that overall simulated rates of respiration were strongly related to observations (N = 180, R^2^ = 0.69, P≤0.01), and that simulated rates of respiration averaged 1±17% lower than observed rates (data not shown). However, the relative differences ([observations-simulations]/observations) varied over time (Text S2). The first two axes of the principal components analysis explained 54% of the variation in the relative differences between observed and simulated respiration and litter chemical characteristics (Figure 6a). Differences in rates between days 10–21 were more strongly related to axis 2, along with litter LCI, KL/AX and ∑Sug. In contrast, differences in respiration for most days ≥50 were more strongly associated with axis 1, along with several chemical characteristics, the strongest being arabinans, AX, galactose, NDF and pCA (Figure 6a).

In contrast to the daily respiration rates, there was no significant relationship between simulated and observed peak respiration rates (means = 17.5±1.8 and 21.4±9.9 mgC·kg soil^−1^·d^−1^, respectively; not shown). The PCA showed that relative differences between peak rates (PR) were more closely related to axis 2 and opposite those of rates between days 10–21 (Figure 6a), with significant positive correlations with KL and KL/AX.

Simulated values of cumulative CO_2_ efflux were also strongly related to observations (N = 180, R^2^ = 0.923, P≤0.01), and averaged only 3±18% greater than observations. The first two axes of the PCA explained 69% of the variation in the relative differences between observed and simulated cumulative CO_2_ efflux and initial litter chemistry characteristics (Figure 6b). All values for CO_2_ efflux were tightly clustered and closely associated with the first axis. Litter chemical characteristics, NDF, arabinans, galactose and AX, were also closely related to the first axis. The relative differences between observations and simulations at day 36 (d36) showed a significant, positive correlation with the initial soluble content of litter (C_1_ = C-SOL) and negative correlation with galactan (GAL) content. Differences by day 59 were also related to C_1_ and galactan. Although there were no significant differences between simulations and observations at day 112, these variations were significantly related to several aspects of initial litter chemistry, including C_1_.

Estimates of microbial production by day 112 for these 12 genotypes ranged 203–303 mgC (mean = 270±25 mgC), and were negatively correlated with KL/AX and final microbial biomass, as well as initial LCI. Production was positively related to total cumulative CO_2_ efflux by day 112 and best-fit values of LCI_T_, as well as initial litter concentrations of Glu, ∑Sug, and C_2_ (all N = 12, P≤0.05). A stepwise regression explained nearly all variation in microbial production as a function of total CO_2_ efflux and initial concentrations of xylans (Xyl) and guaiacyl (G) in litter (N = 12, R^2^ = 0.998, P≤0.01).

## Discussion

### Lignin-cellulose interaction

Simulations provided a close match to observed patterns of holocellulose (C_2_) decay (Figure 4). This pool represented the largest fraction of litter (Table 1), also explaining why simulations closely fit patterns of CO_2_ efflux (Figure 3). Long-term decomposition (months to years) has often been negatively correlated to initial lignin content of litter [3], partly because lignin decays slowly and partly because some products of decomposition increase the size of the non-hydrolysable pool often interpreted as lignin [6]. However, Machinet et al. [11] also demonstrated the effects of biochemical connections between cell wall polysaccharides and lignin on the pattern of cumulative CO_2_ efflux from decomposing maize roots. The fit between k_2_ and LCI (Figure 2a) reveals an interaction between C_2_ and C_3_ in early stages of litter decay [19], well before C_3_ begins to decline [13], [20]. In other words, there is unlikely to be qualitatively separate pools of lignin-shielded and unshielded holocellulose, as is sometimes implied [1], [3]. Instead, biochemical linkages between cellulose, hemicellulose and lignin components of cell walls influence patterns of decomposition throughout the process.

A brief explanation of these relationships is that the structural composition of hemicellulose is a primary chain consisting mainly of xylans with branching arabinan side chains that interact with other cell wall polymers. The level of arabinoxylan substitution (represented by A∶X) increases with the progressive enzymatic degradation of plant material, during both digestion in the rumen [9], [24] and decomposition in soils [12], [13], as the more exposed elements of xylan chains are more readily hydrolyzed than those near arabinan branches. Gunnarsson et al. [25] found that initial xylan and arabinan concentrations in litter were as important as the total amount of hemicellulose in describing C mineralization over the first 9 days of laboratory incubations, and that arabinan was the single most important factor. These results were consistent with those of Machinet et al. [11], who identified initial arabinan content as an early predictor of C mineralization (days 3–7), followed by AX (days 7–14). In addition, hydroxycinnamic acids (ferulic and *p*-coumaric acids) play a key role in cross-linking arabinoxylans with lignin and this cohesive network also hampers decomposition [10], [11], [12]. These cross-linkages explain the negative effect of lignin on holocellulose decay long reported in the literature [1], [3] and also why k_2_ decreased as AX increased early in decomposition (Figure 2).

Our model revision approximated this complex control with a straightforward relationship between LCI and k_2_ (Figure 2) that included interactions between C_2_ and C_3_ (based on empirical sugar and lignin determinations). Moreover, key model parameters LCI_T_ and K_B22_ were most closely related to initial KL/AX ratios of litter, emphasizing the interaction between holocellulose and lignin throughout the decomposition process.

### Soluble dynamics

Data from Machinet et al. [13] also revealed that the soluble C_1_ pool included a fraction (C_1T_) that persisted throughout the study (Figure 4). Although the soluble fraction of litter is usually considered to be highly decomposable, the persistence of a relatively large soluble pool is common in both soils and decaying litter [26], [27]. The soluble pool usually contains a hydrophilic fraction including sugars and amino acids that are readily used by microorganisms [28], [29], and a more hydrophobic portion, including soluble polyphenols (e.g., tannins) that are less rapidly utilized [26], [30]. This differential use of compounds in the C_1_ pool by decomposer microorganisms changes the pool's overall quality and decomposability with time [1]. Although the soluble pool is replenished with degradation products from non-soluble substrates [1], [31], products of C_2_ hydrolysis would enter the more labile fraction of the soluble pool, which cycles much more rapidly than the more persistent fraction [28], [29]. In addition, Machinet et al. [13] found no decrease in the C_3_ pool over time (not shown), so that it's degradation products could not have increased the more persistent fraction of the C_1_ pool (C_1T_). Simulating the persistence of a sizeable pool of C_1_ with GDM required dividing the pool into labile and persistent fractions (Figure 1), with the labile fraction (C_1D_) rapidly declining during decay and the persistent C_1T_ pool remaining intact (Figure 4). Moorhead and Sinsabaugh [16] found that simply routing the products of C_2_ degradation through the whole C_1_ pool GDM could not explain the size of this composite pool.

### Respiration patterns

Simulations tended to overestimate rates of CO_2_ efflux between days 0–14, and underestimate rates between days 14–36 (Figure 3). A positive relationship between the most labile components of litter and early respiration (hours to weeks) or decay rate is commonly reported [32], [33], and most models, including GDM, assume a greater decay rate for soluble substrates [15], [16]. In terms of substrate dynamics, GDM tended to underestimate early losses (day 14) of C_1_ and overestimate C_2_ losses (Figure 4), suggesting that values of k_1max_ should be slightly increased and K_B1i_ (i = 1,2,3) reduced (Table 1), to stimulate decay of C_1D_ and possibly initial CO_2_ efflux. However, GDM underestimated respiration to a greater extent for litter types with lower amounts of C_1D_, i.e., with higher C_1T_ (F292 and F292bm3). We found that C_1T_ was most closely related to the total sugar content of cell walls (∑Sug; N = 4; R^2^ = 0.998; P≤0.05) and that xylan, arabinan and glucan concentrations were highly correlated with each other (not shown). Further resolution of the chemical composition of the soluble fraction (C_1D_ and C_1T_) and its dynamics during decomposition are needed to improve the mathematical descriptions of these relationships.

### Microbial production

Moorhead and Sinsabaugh [16] argued that litter decay is initially limited by microbial action, because there is a time lag in the colonization of fresh litter by decomposer microorganisms (Figure 2). Whether this lag was a numerical (biomass) or functional (physiological) response in the study by Machinet et al. [13] is unknown because they did not monitor microbial biomass. Therefore, the most speculative part of this study was our simulation of microbial dynamics. Nonetheless, the patterns and magnitudes of simulated microbial biomass and turnover rates were consistent with observed patterns of respiration (Figures 3, 5b), reported limits to microbial biomass concentration in soils [21], [22], and changes in litter chemistry likely controlling microbial activities (Figures 2, 4 and 5c; [1]) as well as CUE (Figure 5d) [23]. Parameter estimates for C-assimilation efficiencies (e_i_) for various substrate pools, coefficient of microbial basal respiration rate (g), and maximum biomass∶total system C ratio (BC_max_), most directly affected the relationships between substrate decomposition and biomass dynamics (Table 1). However, variations in any or all of these parameter values generate similar patterns with respect to different litter chemistry, although amplitudes and temporal regimes vary [16].

Our primary reason for simulating biomass dynamics was to determine if litter quality affected microbial production consistent with the idea that more decomposable chemical fractions of litter not only decay more rapidly but also generate more microbial products likely to enter stable soil organic matter pools [4], [5]. In fact, simulated microbial production was significantly and negatively correlated to KL and KL/AX, which are inversely related to decomposability, but production showed no significant positive relationship to any simple measure of litter quality. However, it was positively related to the sum of the two most decomposable carbon pools, i.e., C_2_+C_1D_, (N = 4, rho = 0.982, P≤0.05). The contributions of the relatively larger pools of C_1D_ in litter types F2 and F2bm1 to simulated production were small compared to the larger pools of C_2_ in the other litters, despite the lower C assimilation efficiency of C_2_ versus C_1_ (Table 1). Thus our results were consistent with the observations of Smith et al. [7] and others who found that higher initial rates of C incorporation into biomass coincided with higher losses of litter through respiration [2], [8].

### Sensitivity analysis

The most interesting result of our sensitivity analysis was the lack of any simple, overall interpretation. No single parameter consistently explained>10% of the observed variability in any model behavior (Table 2). Usually a parameter important to explaining one model behavior, such as the contributions of k_1max_ to simulated C_1_ dynamics or cumulative CO_2_ efflux (Table 2), made little contribution to other model outputs. The largest discrepancies between simulations and observations were in early respiration and C_1_ loss (discussed above). Of the tested parameters, e_2_, the C assimilation efficiency for C_2_, appeared to be most important to respiration rates, which seems reasonable because C_2_ was the largest substrate pool and provided most of the C respired (Table 1, Figure 4). As for the dynamics of the C_1_ pool, parameter k_1max_ appeared to be most important, followed by K_B11_; thus parameters controlling the degradation rate of this pool explained differences between simulations and observations (Figure 4). These results are consistent with our earlier conclusion that greater resolution of the C_1_ pool composition and dynamics might provide a better understanding of decomposition.

### Extrapolations

We simulated the decomposition of 12 additional maize genotypes in the second set of incubations conducted by Machinet et al. [11] based on the assumption that the relationships between key parameters, LCI_T_, K_B22_ and C_1T_, and litter chemical characteristics were consistent with those for the four genotypes examined by Machinet et al. [13]. The assumption seemed reasonable because all 16 genotypes were naturally occurring varieties of *Zea mays*, and likely to be more similar in chemistry and tissue architecture than unrelated species more commonly used in comparative decomposition experiments [6].

In general, simulations were within a few percent of observed values of respiration rates and cumulative CO_2_ efflux over the entire period of incubation. Analyses of these differences between simulations and observations provided relatively little additional insight to patterns of cumulative CO_2_ efflux (Figure 6b). The importance of the initial C_1_ pool (as both C_1T_ and C_1D_ estimates) to cumulative CO_2_ efflux through time again emphasized the importance of initial decay rate to longer-term patterns [7], [8]. The relationships between CO_2_ efflux on day 112 and initial arabinan and *p*-coumaric acid concentrations and AX suggest that the cross-linkages among hemicellulose and lignin became increasingly important with progressive decay. The tight cluster of cumulative CO_2_ efflux along the first axis of our PCA also underscored the importance of cross-linkages among cell wall constituents (e.g., arabinan, AX, NDF and *p*-coumaric acid) to litter decay (Figure 6b), consistent with Machinet et al. [11].

The differences between simulated and observed respiration rates were more variable, suggesting temporally shifting controls on decomposition. Peak rates and those on days 14–28 were related to initial KL and KL/AX, as well as glucan and S∶G (Figure 6a). In contrast, rates on days>42 were more closely associated with arabinan, AX, *p*-coumaric acid and ester-linked ferrulic acids, perhaps because polysaccharide-ester linked ferulic acids can form ether-links with lignin [34], [35] and the syringyl units of lignin can be esterified by *p*-coumaric acids, which is typical of grass cell walls [11]. However, the biggest differences between simulated and observed rates were on days 36–42, which were most closely related to KL, ether-linked ferrulic acids and galactan (Figure 6b). The frequent importance of KL and AX (or their chemical constituents) to these patterns was surprising, because KL (in LCI) was used to estimate k_2_, and KL/AX to estimate LCI_T_ and K_B22_ (previously discussed) used in simulations. Clearly, simple linear relationships were insufficient to capture the subtleties of these controls. The relationships between respiration and galactan and C∶N ratio (Figure 6b) hint at a microbial control [14], in part because galactan is sometimes used as an index to microbial contributions [36], but it is also a hemicellulosic sugar, along with arabinan, rhamnan, and xylan [37].

## Conclusions

We found that the level of arabinan substitution in xylan chains (AX) was an important control in early stages of decomposition, and was also linearly related to LCI calculated on the basis of fine scale cell wall chemistry. These relationships provide a plausible, mechanistic explanation for earlier, empirical descriptions of LCI effects on decomposition as a result of biochemical cross-linkages between polysaccharides and lignin. Thus lignin and LCI serve as convenient, negative proxies for the decomposability of litter even at the start of decay. However, additional research is needed to determine the chemical composition and dynamics of the non-hydrolysable product of proximate C analysis that is typically termed “lignin” if we are to discover the mechanistic relationships between LCI and latter stages of decomposition.

We also found that dividing the soluble pool of litter (C_1_) into separate persistent (C_1T_) and labile (C_1D_) pools, was necessary to accurately simulate the dynamics of the composite soluble pool during early decomposition because not all soluble compounds are equally decomposable. The finer scale chemical composition and dynamics of the soluble component of litter is needed to determine the possible sources and fates of these compounds.

These relationships between simulated patterns of litter decay, litter chemistry (LCI, AX, C_1T_ and C_1D_) and microbial productivity were consistent with the notion that the more rapid utilization of substrates with high carbon use efficiency generates greater amounts of microbial products that can contribute to stable soil organic matter pools than more persistent substrates with lower C-assimilation, like lignin. Our assumption that lignin decay provides little to no net C-acquisition by microorganisms is also consistent with recent observations that little lignin C enters stable SOC pools.

Finally, our extrapolations with litter types that differed in initial chemistry demonstrated that relationships we found between key model parameters, LCI_T_, K_B22_ and C_1T_, and decomposition were robust across a large range of maize litter types, highlighting the importance of access (K_B22_) to the largest, rapidly decaying pool of substrate (C_2_) by microorganisms able to use this resource (G_2_), as well as negative controls imposed by the less-accessible substrate pools (KL, C_1T_). Moreover, differences between simulations and observations indicated that temporal controls on decay rates shifted from relative substrate pool sizes (both accessible and persistent) dominating at the start to factors related to cross-linkages between structural polysaccharides and lignin with progressive decomposition.

In closing, our results suggest that interactions between decomposer microorganisms and litter quality characteristics at the earlier stages of decomposition may provide more insights to soil organic C stabilization than later stages dominated by the more persistent chemical characteristics of the cell wall, if indeed microbial products comprise a large fraction of stable soil C pools.

## Supporting Information

Data S1 Data used to test and refine model. Observed respiration rates and chemical composition of residues of maize roots during decomposition [13].
(DOCX)

Figure S1 Differences over time between observed and simulated respiration rates and cumulative CO_2_ efflux from the decomposition of *Zea mays* roots. Relative differences between observations and simulations of litter decomposition from 12 novel maize genotypes [11] for: a. respiration rates (mgC·kg soil^−1^·d^−1^) over time (means±95% confidence intervals, all N = 12), b. cumulative CO_2_ efflux (mgC·kg soil^−1^) over time (means±95% confidence intervals, all N = 12).
(TIFF)

Text S1 Lignocellulose controls. Relationships between decay rate coefficients for polysaccharides (C_2_) and polyphenolics (C_3_) as functions of lignocellulose index (LCI = C_3_/[C_2_+C_3_])).
(DOCX)

Text S2 Sensitivity analysis. Patterns of differences between observed and modeled patterns of respiration associated with decomposition of decaying maize roots.
(DOCX)
