# Supplementary material for: Interacting Microbe and Litter Quality Controls on Litter Decomposition: A Modeling Analysis
Source: PLoS One. 2014 Sep 29;9(9):e108769. doi: 10.1371/journal.pone.0108769 (PMC4181322; doi:10.1371/journal.pone.0108769)
Supplement: Data S1 — Data used to test and refine model. Observed respiration rates and chemical composition of residues of maize roots during decomposition [13]. (DOCX) [file pone.0108769.s001.docx]

Data reported by Machinet et al. [13] used to test and refine the Guild Decomposition Model of Moorhead and Sinsabaugh [16] are included below. The first table includes the maize root carbon pool sizes over time, calculated from detailed chemical analyses described in the text. The second table reports the CO_2_ efflux rates from laboratory incubations of maize roots during decomposition.

Table S1. Chemical fractions of mass (mg C · kg soil^-1^) of four genotypes of maize root litter remaining over time: C_1_ = Van Soest soluble, C_2_ = total cell wall sugars (Gal + Glu + Xyl + Ara + Ac Glu + Ac Gal + Fuc + Rham + Mam), and C_3_ = Klason lignin. Each value is the average of two observations (calculated from Machinet et al. [13]).

| Genotype | Days | C_1_ | C_2_ | C_3_ |
| --- | --- | --- | --- | --- |
| F2 | 0 | 398.8 | 1166.5 | 434.7 |
|  | 14 | 234.1 | 1106.1 | 478.6 |
|  | 36 | 248.6 | 795.4 | 480.7 |
|  | 57 | 233.6 | 689.0 | 473.0 |
|  | 112 | 182.9 | 602.6 | 470.3 |
| F292 | 0 | 391.0 | 1237.1 | 371.9 |
|  | 14 | 274.8 | 1087.1 | 380.3 |
|  | 36 | 274.9 | 731.7 | 379.1 |
|  | 57 | 267.5 | 614.0 | 364.9 |
|  | 112 | 225.4 | 497.6 | 361.1 |
| F292bm3 | 0 | 323.2 | 1311.8 | 365.0 |
|  | 14 | 335.3 | 1051.0 | 343.5 |
|  | 36 | 332.5 | 683.6 | 349.4 |
|  | 57 | 294.5 | 569.6 | 362.9 |
|  | 112 | 285.6 | 451.6 | 343.0 |
| F2bm1 | 0 | 463.5 | 1077.4 | 459.1 |
|  | 14 | 262.0 | 1086.3 | 529.9 |
|  | 36 | 266.8 | 865.0 | 543.4 |
|  | 57 | 275.1 | 775.4 | 513.9 |
|  | 112 | 218.7 | 689.2 | 543.0 |

Table S2. Carbon efflux rate (mg C·kg soil^-1^·d^-1^) during decomposition of four genotypes (F2, F2bm1, F292 and F292bm3) of maize roots over time. Each value is the average of two observations (calculated from Machinet et al. [13]).

| Day | F2 | F2bm1 | F292 | F292bm3 |
| --- | --- | --- | --- | --- |
| 3 | 7.21 | 6.39 | 9.28 | 7.99 |
| 7 | 13.86 | 8.89 | 18.96 | 17.55 |
| 10 | 13.69 | 9.58 | 20.60 | 24.84 |
| 14 | 15.76 | 9.58 | 23.10 | 25.39 |
| 21 | 16.47 | 10.36 | 21.70 | 22.76 |
| 29 | 13.88 | 9.68 | 16.18 | 16.23 |
| 36 | 9.69 | 7.57 | 10.72 | 10.72 |
| 42 | 7.64 | 6.39 | 8.32 | 8.32 |
| 51 | 6.11 | 5.32 | 6.56 | 6.50 |
| 57 | 4.70 | 4.11 | 5.06 | 5.01 |
| 70 | 3.78 | 3.15 | 4.21 | 3.93 |
| 80 | 2.78 | 2.27 | 3.23 | 2.91 |
| 87 | 2.56 | 2.00 | 2.95 | 2.68 |
| 95 | 1.80 | 1.43 | 2.19 | 1.91 |
| 112 | 1.80 | 1.43 | 2.18 | 1.92 |
