# Supplementary figures and images for: Interacting Microbe and Litter Quality Controls on Litter Decomposition: A Modeling Analysis
Source: PLoS One. 2014 Sep 29;9(9):e108769. doi: 10.1371/journal.pone.0108769 (PMC4181322; doi:10.1371/journal.pone.0108769)

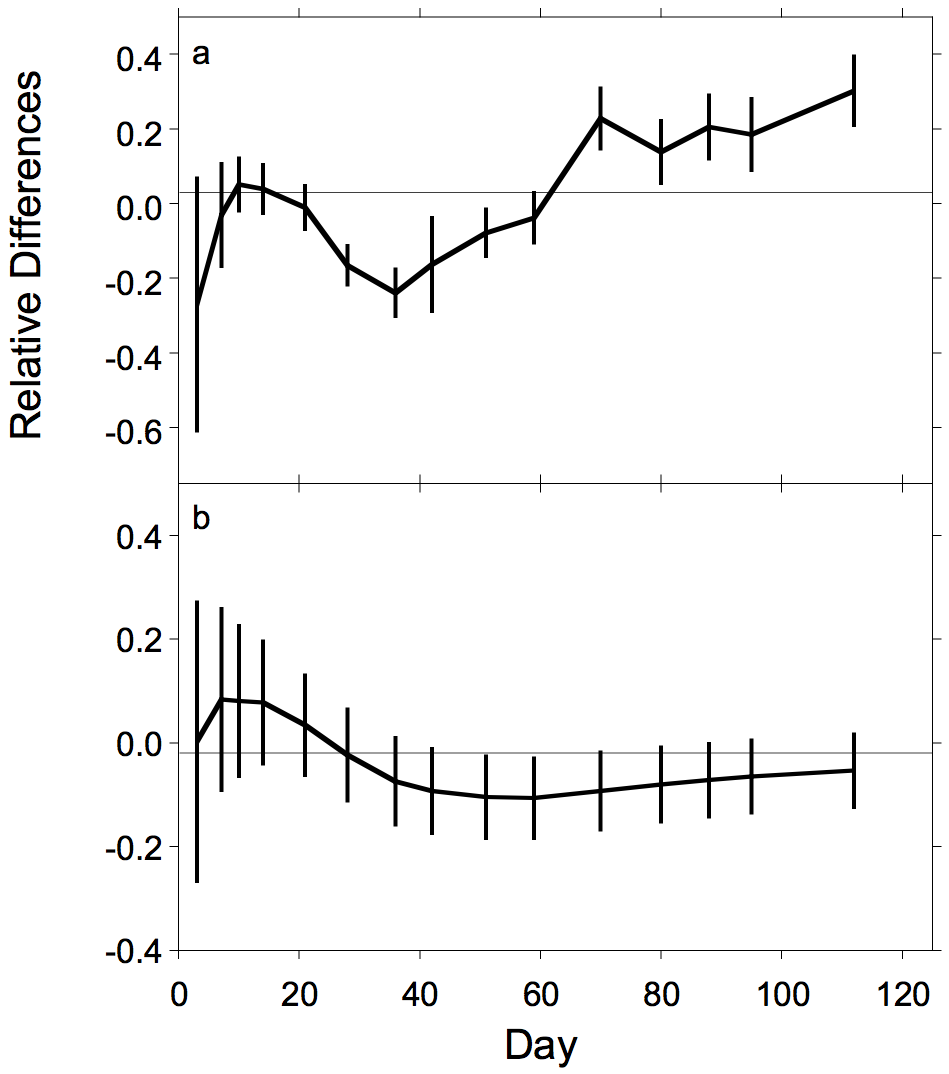

Supplement: Figure S1 — Differences over time between observed and simulated respiration rates and cumulative CO2 efflux from the decomposition of Zea mays roots. Relative differences between observations and simulations of litter decomposition from 12 novel maize genotypes [11] for: a. respiration rates (mgC·kg soil−1·d−1) over time (means±95% confidence intervals, all N = 12), b. cumulative CO2 efflux (mgC·kg soil−1) over time (means±95% confidence intervals, all N = 12). (TIFF) [file pone.0108769.s002.tiff]
