# Supplementary material for: Interacting Microbe and Litter Quality Controls on Litter Decomposition: A Modeling Analysis
Source: PLoS One. 2014 Sep 29;9(9):e108769. doi: 10.1371/journal.pone.0108769 (PMC4181322; doi:10.1371/journal.pone.0108769)
Supplement: Text S1 — Lignocellulose controls. Relationships between decay rate coefficients for polysaccharides (C2) and polyphenolics (C3) as functions of lignocellulose index (LCI = C3/[C2+C3])). (DOCX) [file pone.0108769.s003.docx]

**Text S1. Lignocellulose Controls**

Moorhead et al. [19] described the decay rate coefficient (k_3_) for C_3_ as a linear function of LCI, i.e., k_3_ = m_3_·LCI + k_3max_, given an empirically observed maximum value (k_3max_) and slope (m_3_; [20]). This relationship generates k_3_ = 0 at a threshold level of LCI = LCI_T_ (ca. LCI = 0.4) and k_3_ = k_3max_ at LCI = 0.7 [16, 20], so that m_3_ = k_3max_/(0.7-LCI_T_). The decay rate coefficient (k_2_) for C_2_ was calculated as two linear functions of C_3_ [19]. The first described k_2_ when LCI ≥ LCI_T_, i.e., k_2_ = m_3_·e_2_/e_3_·(LCI-0.7)+k_3max_, such that k_2_ = k_3max_ when LCI = 0.7, and e_2_ and e_3_ are the assimilation coefficients for C released from C_2_ and C_3_, respectively. Finally, k_2_ = m_2_·LCI+k_2max_ when LCI ≤ LCI_T_, in which slope m_2_ is the difference between k_2_ = k_2max_ at LCI = 0 and k_2_ = m_3_·e_2_/e_3_·(LCI_T_-0.7)+k_3max_ at LCI = LCI_T_, divided by the difference in LCI values 0.7 and LCI_T_.
