# Supplementary material for: Interacting Microbe and Litter Quality Controls on Litter Decomposition: A Modeling Analysis
Source: PLoS One. 2014 Sep 29;9(9):e108769. doi: 10.1371/journal.pone.0108769 (PMC4181322; doi:10.1371/journal.pone.0108769)
Supplement: Text S2 — Sensitivity analysis. Patterns of differences between observed and modeled patterns of respiration associated with decomposition of decaying maize roots. (DOCX) [file pone.0108769.s004.docx]

**Text S2. Sensitivity Analysis**

Simulated respiration rates exceeded observations by a maximum of 24% on day 36, when observations averaged 8.41±0.98 mgC·kg soil^-1^·d^-1^, and underestimated rates by a maximum of 30% on day 112, when observations averaged 2.43±0.52 mgC·kg soil^-1^·d^-1^ (Figure S3a). Simulations overestimated cumulative CO_2_ efflux by a maximum of 11% on day 59, when observations averaged 699 mgC·kg soil^-1^ (N = 12, P≥0.05), but no significant differences existed between observations and simulations before day 42 or after day 80 (Figure S3). Indeed, simulations significantly overestimated cumulative CO_2_ efflux between days 42-81, probably because they overestimated CO_2_ efflux rates between days 36-42. Curiously, these patterns were opposite those observed for the best-fit model simulations of the four initial genotypes (Figure 3).
